# Supplementary material for: HDL in COVID-19 Patients: Evidence from an Italian Cross-Sectional Study
Source: J Clin Med. 2021 Dec 18;10(24):5955. doi: 10.3390/jcm10245955 (PMC8708284; doi:10.3390/jcm10245955)
Supplement: Supplementary file 1 [file jcm-10-05955-s001.zip › jcm-1458475-supplementary.pdf]

Supplemental Tables

Supplementary Table S1. Associations between triglycerides and white blood cells in COVID-19 patients, univariate linear regression models.

	β	SE	95% CI		P-value
**White blood cells**	**0.004**	**0.002**	**0.001**	**0.007**	**0.0124**
**Lymphocytes**	**0.008**	**0.003**	**0.002**	**0.014**	**0.0086**
**Basophils**	**0.602**	**0.247**	**0.118**	**1.087**	**0.0148**
**Monocytes**	0.030	0.020	-0.010	0.070	0.1369
**Eosinophils**	0.080	0.058	-0.034	0.193	0.1678
**Neutrophils**	0.002	0.002	-0.001	0.006	0.2452

Supplementary Table S2. Descriptive statistics of white blood cells count in COVID-19 patients.

	Mean	STD	Median	Q1	Q3
**WBC, x10^3/μL**	8143.90	3853.20	7610.00	5340.00	10250.00
**Neutrophils, x10^3/μL**	6346.57	3290.49	5840.00	3680.00	8290.00
**Lymphocytes, x10^3/μL**	1253.14	1933.42	870.00	640.00	1220.00
**Monocytes, x10^3/μL**	474.19	299.58	420.00	280.00	600.00
**Eosinophils, x10^3/μL**	49.62	105.12	10.00	0.00	60.00
**Basophils, x10^3/μL**	20.77	24.32	10.00	10.00	20.00

STD, standard deviation; Q, quartile.
